# Supplementary material for: Role and mechanism of NCAPD3 in promoting malignant behaviors in gastric cancer
Source: Front Pharmacol. 2024 Apr 22;15:1341039. doi: 10.3389/fphar.2024.1341039 (PMC11070777; doi:10.3389/fphar.2024.1341039)
Supplement: Supplementary file 11 [file DataSheet2.ZIP › GSEA/immunologic/my_analysis.Gsea.1599462003817/GSE21063_3H_VS_16H_ANTI_IGM_STIM_BCELL_DN.html]

Details for gene set GSE21063\_3H\_VS\_16H\_ANTI\_IGM\_STIM\_BCELL\_DN[GSEA]

|  || Dataset | filtered\_dataset.sample\_info.cls#WT\_versus\_NCAPD3\_MUT |
| Phenotype | sample\_info.cls#WT\_versus\_NCAPD3\_MUT |
| Upregulated in class | WT |
| GeneSet | GSE21063\_3H\_VS\_16H\_ANTI\_IGM\_STIM\_BCELL\_DN |
| Enrichment Score (ES) | 0.39683354 |
| Normalized Enrichment Score (NES) | 1.8530443 |
| Nominal p-value | 0.0050933785 |
| FDR q-value | 0.6503317 |
| FWER p-Value | 1.0 |
Table: GSEA Results Summary

  

Fig 1: Enrichment plot: GSE21063\_3H\_VS\_16H\_ANTI\_IGM\_STIM\_BCELL\_DN      
 Profile of the Running ES Score & Positions of GeneSet Members on the Rank Ordered List

  

| SYMBOL | TITLE | RANK IN GENE LIST | RANK METRIC SCORE | RUNNING ES | CORE ENRICHMENT || 1 | 51111 | KMT5B | 2 | 1.217 | 0.0770 | Yes |
| 2 | 23272 | FAM208A | 20 | 1.032 | 0.1312 | Yes |
| 3 | 222236 | NAPEPLD | 36 | 0.977 | 0.1833 | Yes |
| 4 | 80746 | TSEN2 | 53 | 0.909 | 0.2303 | Yes |
| 5 | 26272 | FBXO4 | 133 | 0.767 | 0.2227 | Yes |
| 6 | 81790 | RNF170 | 141 | 0.757 | 0.2665 | Yes |
| 7 | 7248 | TSC1 | 158 | 0.718 | 0.3012 | Yes |
| 8 | 54919 | DNAAF5 | 184 | 0.690 | 0.3276 | Yes |
| 9 | 23288 | IQCE | 186 | 0.688 | 0.3712 | Yes |
| 10 | 84142 | FAM175A | 211 | 0.667 | 0.3968 | Yes |
| 11 | 84874 | ZNF514 | 283 | 0.606 | 0.3846 | No |
| 12 | 5465 | PPARA | 375 | 0.550 | 0.3544 | No |
| 13 | 9202 | ZMYM4 | 427 | 0.516 | 0.3508 | No |
| 14 | 8187 | ZNF239 | 440 | 0.508 | 0.3749 | No |
| 15 | 57506 | MAVS | 507 | 0.470 | 0.3576 | No |
| 16 | 54951 | COMMD8 | 600 | 0.423 | 0.3184 | No |
| 17 | 10274 | STAG1 | 633 | 0.406 | 0.3214 | No |
| 18 | 79807 | GSTCD | 644 | 0.402 | 0.3402 | No |
| 19 | 57679 | ALS2 | 656 | 0.399 | 0.3579 | No |
| 20 | 79726 | WDR59 | 725 | 0.361 | 0.3321 | No |
| 21 | 23294 | ANKS1A | 768 | 0.339 | 0.3236 | No |
| 22 | 4285 | MIPEP | 815 | 0.307 | 0.3102 | No |
| 23 | 516 | ATP5G1 | 826 | 0.301 | 0.3223 | No |
| 24 | 6732 | SRPK1 | 835 | 0.291 | 0.3353 | No |
| 25 | 9204 | ZMYM6 | 1101 | -0.477 | 0.1747 | No |
| 26 | 283232 | TMEM80 | 1321 | -0.740 | 0.0643 | No |
Table: GSEA details [plain text format]

  

Fig 2: GSE21063\_3H\_VS\_16H\_ANTI\_IGM\_STIM\_BCELL\_DN      
 Blue-Pink O' Gram in the Space of the Analyzed GeneSet

  

Fig 3: GSE21063\_3H\_VS\_16H\_ANTI\_IGM\_STIM\_BCELL\_DN: Random ES distribution      
 Gene set null distribution of ES for **GSE21063\_3H\_VS\_16H\_ANTI\_IGM\_STIM\_BCELL\_DN**
